# Supplementary material for: Five-year fertilization alters soil microbial composition and functionality in sandy grassland
Source: Microbiol Spectr. 2026 Apr 14;14(5):e02963-25. doi: 10.1128/spectrum.02963-25 (PMC13141923; doi:10.1128/spectrum.02963-25)
Supplement: Supplemental tables and figures — Tables S1 and S2, and Figures S1 to S6. [file spectrum.02963-25-s0001.doc]

**Supporting Information for**

**Five-year fertilization alters soil microbial composition and functionality in sandy grassland**

Rui Zhang ^a, b, c, d^, Yulin Li ^a, c^, Xueyong Zhao ^a, b^, A. Allan Degen ^e^, Xinping Liu ^a, c^, Jie Lian ^a, c^, Yuqiang Li ^a, c *^, Yalin Wu ^a, b, c^, Zhanhuan Shang ^f^

^a^ Inner Mongolia Naiman Agroecosystem National Field Observation and Research Station, State Key Laboratory of Ecological Safety and Sustainable Development in Arid Lands, Northwest Institute of Eco-Environment and Resources, Chinese Academy of Sciences, Lanzhou 730000, China

^b^ Urat Desert-grassland Research Station, Northwest Institute of Eco-Environment and Resources, Chinese Academy of Sciences, Lanzhou 730000, China

^c^ University of Chinese Academy of Sciences, Beijing 100049, China

^d^ Key Laboratory of Stress Physiology and Ecology in Cold and Arid Region of Gansu Province, Lanzhou 730000, China

^e^ Blaustein Institutes for Desert Research, Ben-Gurion University of Negev, Beer Sheva 8410500, Israel

^f^ College of Ecology, Lanzhou University, Lanzhou 730000, China

* Corresponding authors.

E-mail addresses: liyq@lzb.ac.cn (Y.Q. Li).

Tel/Fax: 0086-931-4967219.

**Contents of this file**

**Supplementary Tables S1-S2, and Figures S1-S6**

**Tables S1-S2**

**Table S1 The four fertilizer treatments applied each year from 2018**

| Treatments | before sowing maize | the maize jointing stage |
| --- | --- | --- |
| CK | - | - |
| CF | 44.4 g (NH_4_)_2_HPO_4_/m^2^ | 59.3 g CO(NH_2_)_2_/m^2^ |
| M | 0.006 m^3^ cow dung/m^2^ | - |
| CF_M | 44.4 g (NH_4_)_2_HPO_4_/m^2^  + 0.006 m^3^ cow dung/m^2^ | 59.3g CO(NH_2_)_2_/m^2^ |

Note: CK= No fertilizer; CF = Chemical fertilizer; M= Manure; CF_M = Chemical fertilizer + Manure.

Table S2 Effect of fertilization on soil physico-chemical properties and above-ground biomass (means ± SD, n = 5)

| Treatment | SG | CK | CF | M | CF_M |
| --- | --- | --- | --- | --- | --- |
| SWC (%) | 3.08±1.13 ^b^ | 4.10±0.89 ^ab^ | 3.95±0.84 ^ab^ | 4.88±0.86 ^a^ | 5.12±1.27 ^a^ |
| pH | 7.46±0.15 ^b^ | 7.87±0.08 ^a^ | 7.53±0.05 ^b^ | 7.76±0.16 ^a^ | 7.73±0.08 ^a^ |
| EC (μScm^-1^) | 36.9±3.43 ^a^ | 32.1±1.78 ^b^ | 32.4±1.14 ^b^ | 35.2±2.73 ^ab^ | 38.3±3.16 ^a^ |
| SBD (g cm^-3^) | 1.58±0.07 ^a^ | 1.58±0.06 ^a^ | 1.57±0.04 ^a^ | 1.51±0.01 ^ab^ | 1.49±0.05 ^b^ |
| SC (g kg^-1^) | 2.33±0.68 ^b^ | 2.12±0.38 ^b^ | 2.60±0.83 ^b^ | 4.00±0.58 ^a^ | 3.79±0.69 ^a^ |
| TN (g kg^-1^) | 0.22±0.05 ^b^ | 0.18±0.01 ^b^ | 0.23±0.04 ^b^ | 0.35±0.05 ^a^ | 0.34±0.06 ^a^ |
| AVP (mg kg^-1^) | 25.2±9.21^ab^ | 18.8±4.60 ^b^ | 26.1±8.20 ^ab^ | 33.6±5.18 ^a^ | 33.0±0.87 ^a^ |
| AVN (mg kg^-1^) | 40.0±7.61 ^b^ | 30.7±4.72 ^c^ | 36.5±4.51 ^bc^ | 50.8±4.83 ^a^ | 56.1±8.30 ^a^ |
| AGB (g m^-2^） | 141±46.4 ^c^ | 990±297.1 ^b^ | 1886±838.7 ^a^ | 1664±406.9 ^ab^ | 2405±1015.4 ^a^ |

Note: SG=Sandy grassland; CK= No fertilizer; CF = Chemical fertilizer addition; M= Manure addition; CF_M = Chemical fertilizer + Manure addition. SWC = Soil water content (%); EC = Electrical conductivity; SBD = Soil bulk density; SC = Total soil carbon; TN = Total soil nitrogen; AVP = Available phosphors; AVN = Available nitrogen; AGB = Above-ground biomass. Means with different letters within a row differ from each other (*p* < 0.05).

**Figures S1-S6**

**
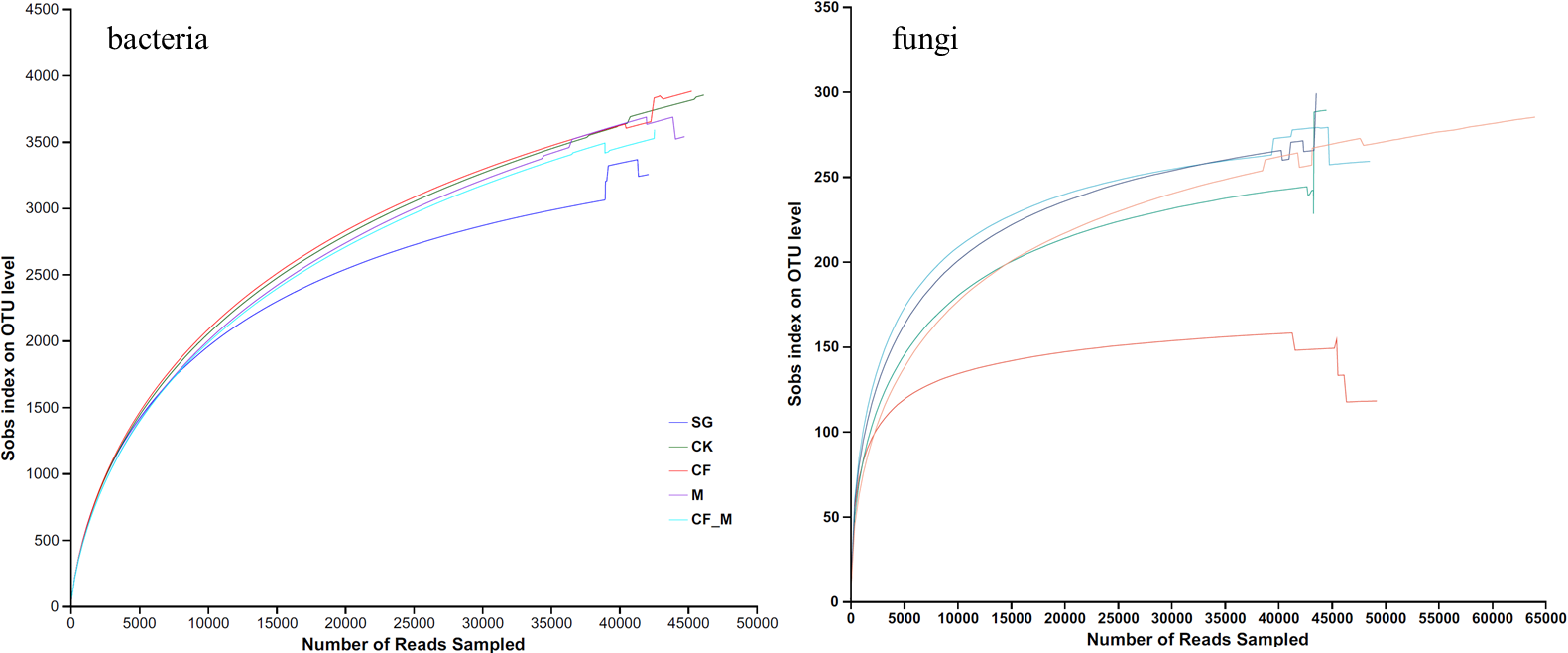
**

Figure S1 The rarefaction curves of soil microbiota in the samples. SG = Sandy grassland; CK= No fertilizer; CF = Chemical fertilizer; M = Manure; CF_M = Chemical fertilizer + Manure.

**
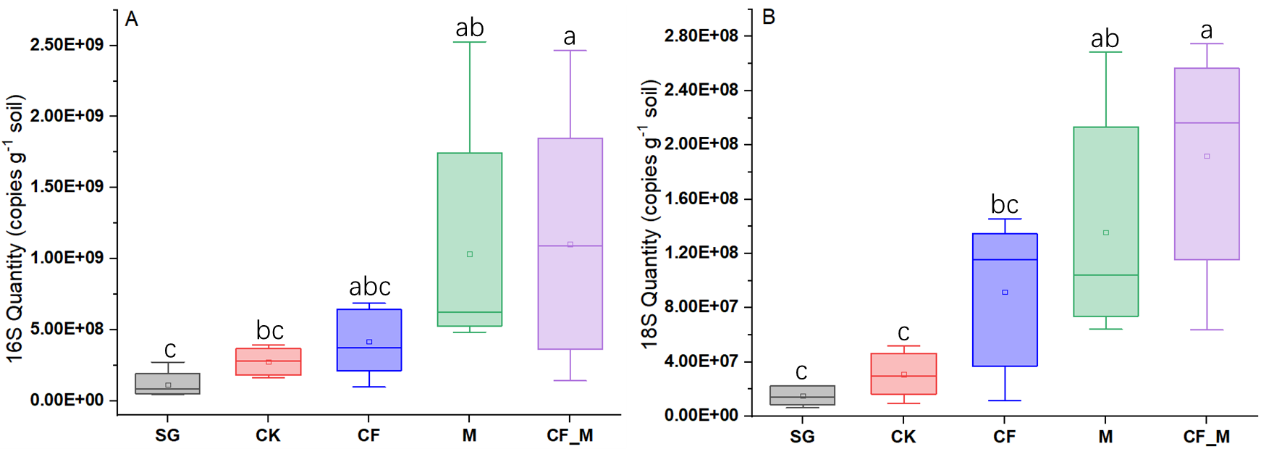
**

Figure S2 Bacterial 16S rRNA gene (A) and fungal 18S rRNA gene (B) copy numbers as determined by quantitative polymerase chain reaction (qPCR). Means with different letters differ from each other (*p <* 0.05). SG = Sandy grassland; CK= No fertilizer; CF = Chemical fertilizer; M = Manure; CF_M = Chemical fertilizer + Manure.

**
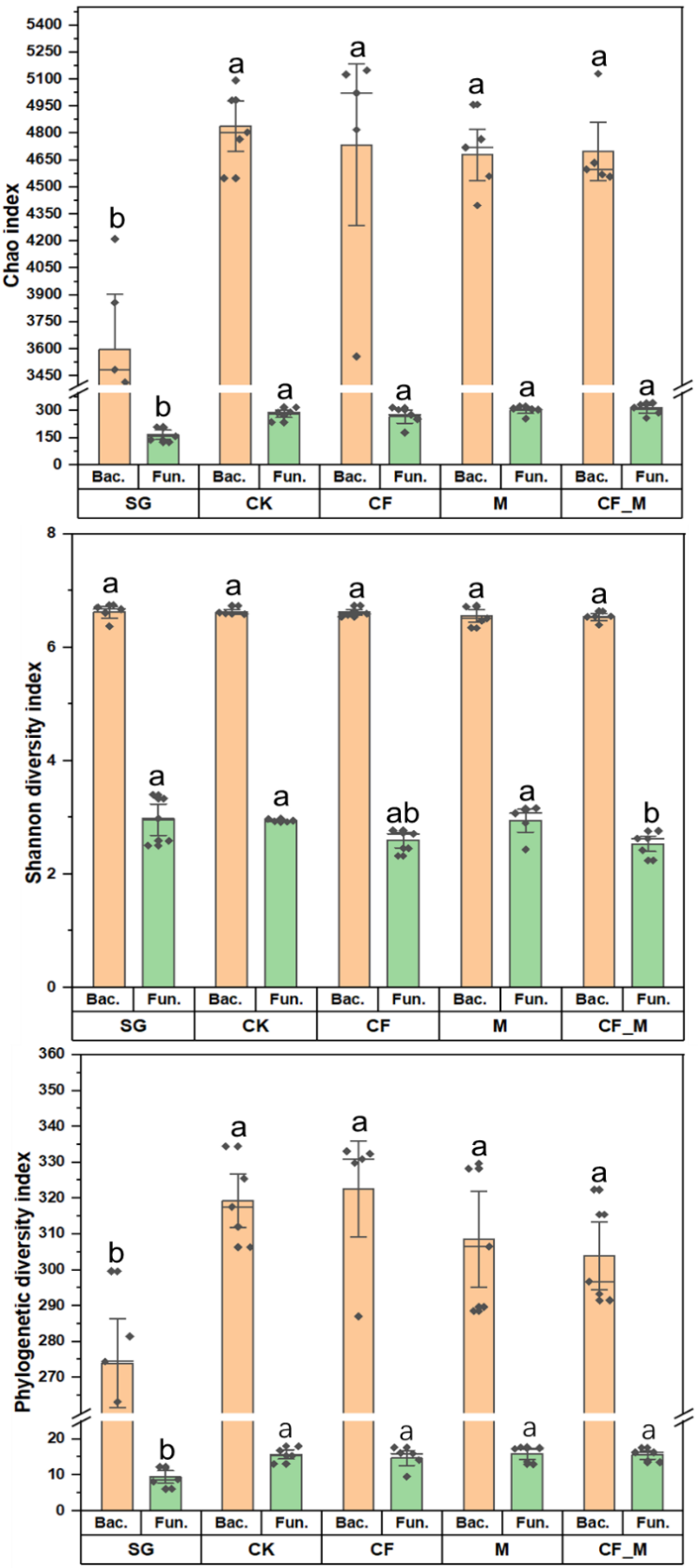
**

Figure S3 Soil bacterial and fungal Chao and Shannon diversity indices with the fertilizer treatments. Means with different letters within bacteria (Bac.) and within fungi (Fun.) in each index differ from each other (*p* < 0.05). SG = Sandy grassland; CK = No fertilizer; CF = Chemical fertilizer; M = Manure; CF_M = Chemical fertilizer + Manure.

**
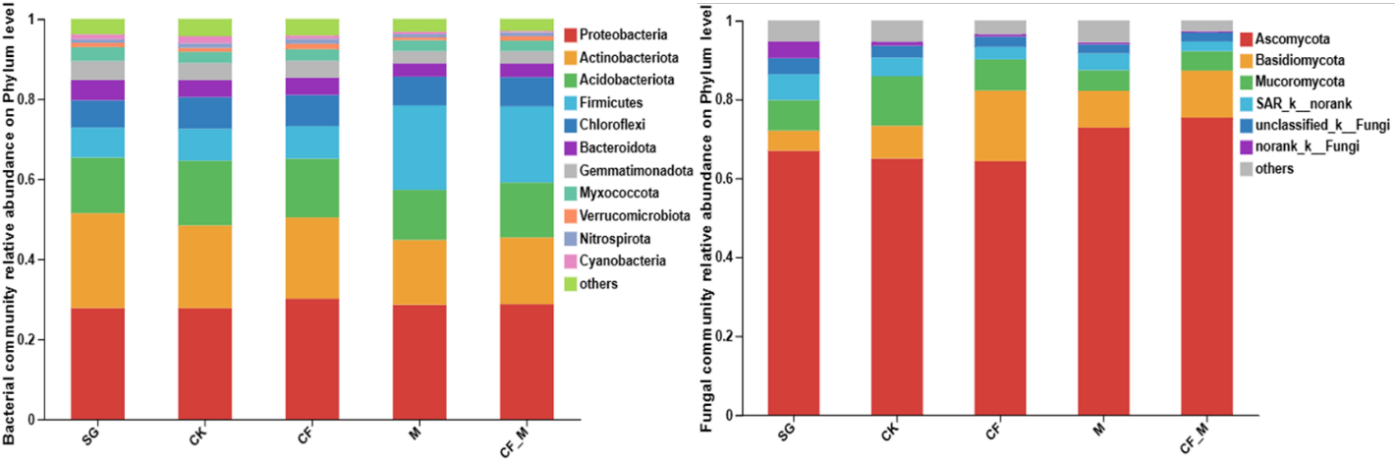
**

Figure S4 Relative abundances of bacteria (left) and fungi (right) at the phylum level with different fertilizer treatments. SG = Sandy grassland; CK = No fertilizer; CF = Chemical fertilizer; M = Manure; CF_M = Chemical fertilizer + manure.


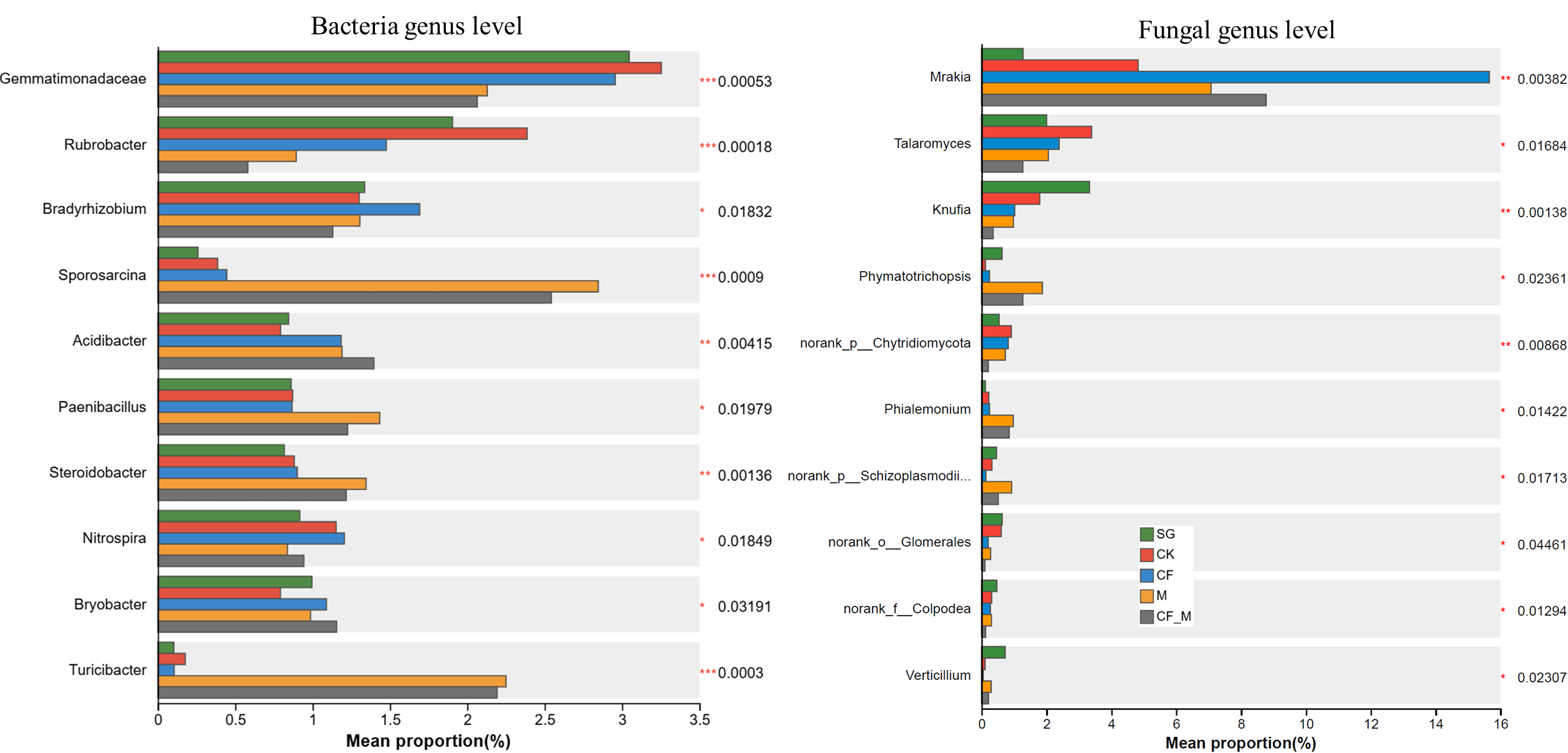


Figure S5 Relative abundances of soil bacteria (left) and fungi (right) among treatments at the genus level. SG = Sandy grassland; CK = No fertilizer; CF = Chemical fertilizer; M = Manure; CF_M = Chemical fertilizer + manure. ****p* < 0.001; ***p* < 0.01; **p* < 0.05.


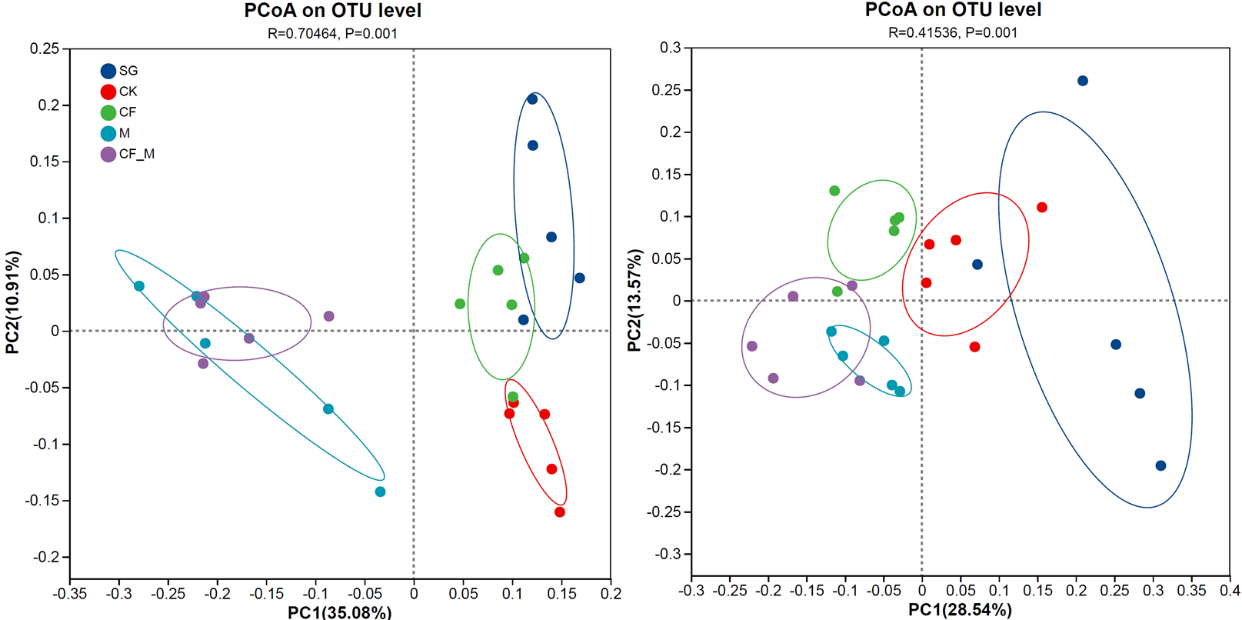


Figure S6 The dissimilarity of bacteria (left) and fungi (right) at the OTU level with different fertilizer treatments. SG = Sandy grassland; CK = No fertilizer; CF = Chemical fertilizer; M = Manure; CF_M = Chemical fertilizer + manure.
